# Cellular Dynamics of Transition Metal Exchange on Proteins: A Challenge but a Bonanza for Coordination Chemistry

**DOI:** 10.3390/biom10111584

**Published:** 2020-11-21

**Authors:** Jean-Marc Moulis

**Affiliations:** 1 Alternative Energies and Atomic Energy Commission—Fundamental Research Division—Interdisciplinary Research Institute of Grenoble (CEA-IRIG), University of Grenoble Alpes, F-38000 Grenoble, France; jmmoulis@protonmail.com; 2 National Institute of Health and Medical Research, University of Grenoble Alpes, Inserm U1055, F-38000 Grenoble, France; 3 Laboratory of Fundamental and Applied Bioenergetics (LBFA), University of Grenoble Alpes, Inserm U1055, F-38000 Grenoble, France

**Keywords:** transition metals, redox activity, regulation, chaperone, metal toxicity, inflammation, specificity, labeling, recombinant technology, iron

## Abstract

Transition metals interact with a large proportion of the proteome in all forms of life, and they play mandatory and irreplaceable roles. The dynamics of ligand binding to ions of transition metals falls within the realm of Coordination Chemistry, and it provides the basic principles controlling traffic, regulation, and use of metals in cells. Yet, the cellular environment stands out against the conditions prevailing in the test tube when studying metal ions and their interactions with various ligands. Indeed, the complex and often changing cellular environment stimulates fast metal–ligand exchange that mostly escapes presently available probing methods. Reducing the complexity of the problem with purified proteins or in model organisms, although useful, is not free from pitfalls and misleading results. These problems arise mainly from the absence of the biosynthetic machinery and accessory proteins or chaperones dealing with metal / metal groups in cells. Even cells struggle with metal selectivity, as they do not have a metal-directed quality control system for metalloproteins, and serendipitous metal binding is probably not exceptional. The issue of metal exchange in biology is reviewed with particular reference to iron and illustrating examples in patho-physiology, regulation, nutrition, and toxicity.

## Foreword

This review deals with the dynamics of transition metals’ homeostasis in the context of, mainly, mammalian cells. Two unrelated points must be clarified at the onset.

First, this review does not pretend to be comprehensive, and only a few examples will illustrate the presented items. Relatively recent references will be quoted, so that the reader can go back in time and details if interested. Only in a few cases, the work of the actual pioneers will be mentioned when it is unambiguously known and attributed by this author.

Second, the word ‘metal’ is too often improperly used, principally in the literature that has little interest in even basic principles of chemistry, hence of biochemistry. Consequently, inorganic elements beyond group 15 of the periodic table (such as arsenic and, worse, selenium) will not be considered here, since their properties are hardly or not at all those of metals. Instead, this review deals with only transition metals, i.e., those with outer *d* orbitals, and mainly the *3d* one among them, which also excludes metals of groups 1 (lithium, sodium), 2 (magnesium, calcium), 13 (aluminum, gallium), and 14 (tin, lead).

## 1. Introduction

It follows from the second point above that transition metals are involved in non-covalent bonds that are generally weaker than the covalent ones involving the elements of (organic) biochemistry, including combined forms of carbon, oxygen, nitrogen, and a few other elements. Therefore, the paraphernalia of high-energy-requiring enzymes supporting the core part of the Chemistry of Life is less developed, although not absent, for the activities dealing with transition metals in biology. However, ionic forms of transition metals build the essential part (very often at the active site) of a wealth of enzymes and proteins. Estimates indicate that at least 25% of the proteomes require metals for function [1,2]. However, as the authors of such estimates indicate, bioinformatics approaches rely on the screening of characteristic metal-binding sites in protein sequences, and identification of metals in structural studies. Furthermore, demonstrating the essential roles of metals in the function of parts of the proteome is not always straightforward. As will be shown below with a few examples, metal binding reactions to proteins exist beyond easily identified ones. It can thus be safely stated that the above proportions of the ‘*metalloproteome*’ are underestimations when considering all reactions in which proteins interact with metals. Pushing these lines of thinking further, it may even be posited in a provocative and largely unsupported statement that seemingly all proteins do bind metal ions in cells, quite often in a fortuitous, silent, and transient way. Indeed, the serendipitous presence of unexpected metals may be observed [3,4] (*vide infra*), and the proteome of selected microorganisms appears largely dominated by metalloproteins in relation with their living environment [5]. A subset of amino acid sidechains, such as those of cysteines, histidines, carboxylic acids (glutamate and aspartate), and a few others in particular local environments, are the main ligands of transition metal cations. Occasionally, the C- and N-termini and the amide group of the peptide bond may participate in the coordination sphere, e.g., [6,7]. The metal-binding sidechains are ubiquitous in proteins (the above listed represent more than 15% of the amino acids in the proteomes of animals), and they very often participate in building proper coordination spheres around transition metal ions if given the opportunity by suitable folding of the protein. In the following, because the boundaries between the family of proteins that tightly binds specific metal compounds at their active site (metalloproteins) and that of proteins interacting with metals in a way or another (metal-protein complexes) leave room for large uncertainties, no differences between them will be made.

When considering the dynamics of metal–protein interactions, attention must be focused on (cat)ionic solutions. Indeed, the state of transition metals relevant in Biology is rarely gas or solid. Exceptions may be found for gaseous mercury or the fumes to which welders may be exposed, and for accidental ingestion of metals or the occurrence of pica disease (metallophagia). However, in all these exposure cases, the ensuing metal toxicity, thus the biological relevance, is due to the released or converted ions, in the acidic stomach of animals after ingestion for instance.

The relative weakness of chemical bonds involving metal cations in biological environments makes ground for a very active dynamics of metal exchange in proteins. In terms of kinetics, transition metal ions in biologically relevant aqueous solutions are minimally surrounded by water molecules and hydroxide ions. To set a very approximate time frame, the rate of substitution of the poor ligand water is generally faster than 10^6^ s^−1^ with ions of transition metals [8]. It means that the replacement of the aqueous coordination sphere of transition metals occurs in a time range that is hardly accessible for most biochemical methods. Such rate constants give only a general idea, and they significantly fluctuate with other ligands, but they indicate that metal–ligand exchange in biochemistry can be fast and transient in many situations. In parallel, the occurrence of a “free” transition metal cation as implying a mere M^n+^ ion (M for a given metal, n for its oxidation state, which is an integer) devoid of any ligand in a biological environment is totally unrealistic and misleading. Let us hope the use of the ‘free metal’ term fades in the future biochemical literature because of its lack of accuracy, although it does not seem to be a strong current tendency.

## 2. Biological Availability of Transition Metals: Not So Easy, even in an Ocean of Plenty

### 2.1. Evolution Could not Get Rid of Transition Metals

The presence of metals in present-day Biochemistry is more than a remnant of the prebiotic world since the most sophisticated living species have developed new activities around metalloproteins and metalloenzymes. For instance, mammalian iron homeostasis significantly differs from the use and regulation of iron found in other organisms. A defensin (hepcidin) plays a pivotal role in systemic iron homeostasis, whereas cellular iron sensing relies on either assembly and destruction of an iron sulfur cluster in Iron Regulatory Protein (IRP) 1 [9], or that of an oxo-bridged binuclear iron center coupled with the redox switch of a [2Fe-2S] cluster in the alternative iron sensor FBXL5 [10]. These features and their associated biochemical networks are absent in other organisms, and they appear as evolutionary recent events involving metals. In all these and other cases, the dynamics of iron exchange on the regulating proteins through biosynthesis and degradation of their clusters, or binding and release of a metal, is a central feature of the molecular mechanism.

### 2.2. Transition Metals vs. Oxygen: Marriage or Divorce?

The dynamics of metal exchange on proteins is obviously directly associated with metal availability. Whereas organic molecules (sugars, lipids, a very wide diversity of substrates for microorganisms, etc.) can be enzymatically processed to provide the biomass and energy needed by cells (metabolism), metals pose specific problems. Most transition metals are now present in the biosphere in a form that is hardly suitable for biological assimilation, for high eukaryotes, such as plants and animals, at least. Indeed, environmental transition metals are mostly oxidized in the present-day oxygen-rich atmosphere, and they are generally poorly soluble in water, except for those forming stable oxyanions, such as molybdate. Yet, multicellular organisms did not find better ways than metal-centered molecules to deal with oxygen and use it: hemoglobin and cytochrome c oxidase are prominent examples. The problem of transition metal oxidation and precipitation was likely absent in the Archean terrestrial conditions under which Life began and relied heavily on transition metals, iron in particular [11]. As photosynthetic oxygen production increased, transition metals got more efficiently oxidized and buried in the lithosphere, a progression in which the Great Oxygenation Event, about 2–2.5 Ga ago, played a prominent role [12]. It follows that present-day living species cope with difficult-to-get but mandatory transition metals.

The preceding paragraphs emphasize that transition metals are mandatory for all life forms, that present-day living cells deploy costly uptake strategies, and that most aspects of cellular metal homeostasis rely on weak bonding and fast exchange. The latter point will be developed in the following with some bias toward examples taken from iron homeostasis and the roles of iron-sulfur proteins since these are topics this author has been active in. However, many of the items reviewed herein and their underlying principles are not restricted to iron, and they have significant bearings for other transition metals. The present review may be considered as a partial update with a different shade of a previously published tutorial [2].

## 3. Getting Metals In and Out of Cells: Metal Exchange in Action

As stated above, getting metals from the environment for cells nowadays is not as straightforward as it might have been in the conditions prevailing in the Archean ages. However, it appears that evolution has been quite active to maintain a sufficient level of metals for cellular purposes despite the increasing cost of obtaining them. For several decades now, knowledge has been increasing on both the means cells develop to extract transition metals from their surroundings, and the often sophisticated pathways leading them to their proper intra-cellular targets.

### 3.1. Metal Exchange through Biological Membranes

For simple forms of transition metal cations, a range of trans-membrane transporters has been characterized. By ‘simple form’, it is meant metal cations with loose ligands. The simplest one may be aquated M^n+^; however, as already indicated, such ions—with exceptions—surrounded by usually six water molecules or hydroxide anions for a complete coordination sphere are neither very stable in oxygenated solutions nor very soluble. Despite this drawback, simple forms of metal cations are available for cells. For instance, copper in mammalian plasma is mainly bound to proteins, such as ceruloplasmin and albumin [13], that deliver it to cells via the copper transporter 1 (CTR1, also know as SLC31A1) uptake system. According to the currently held model for the mechanism of this trimeric transporter [14], plasma protein-bound (cupric) copper is transferred as cuprous ion to the CTR1 ectodomain in which methionine residues that are close in the sequence play an important role. The metal ion then travels through the pore by hopping between transient metal-binding sites in which other methionine and histidine residues are probably involved [15]. The intracytoplasmic domain, which also plays a regulatory role in the transport mechanism, gets the metal for delivery to intracellular targets (chaperones, *vide infra*). Intracellular binding and use of copper likely creates a copper gradient through the membrane that triggers transport.

**Figure 1:**
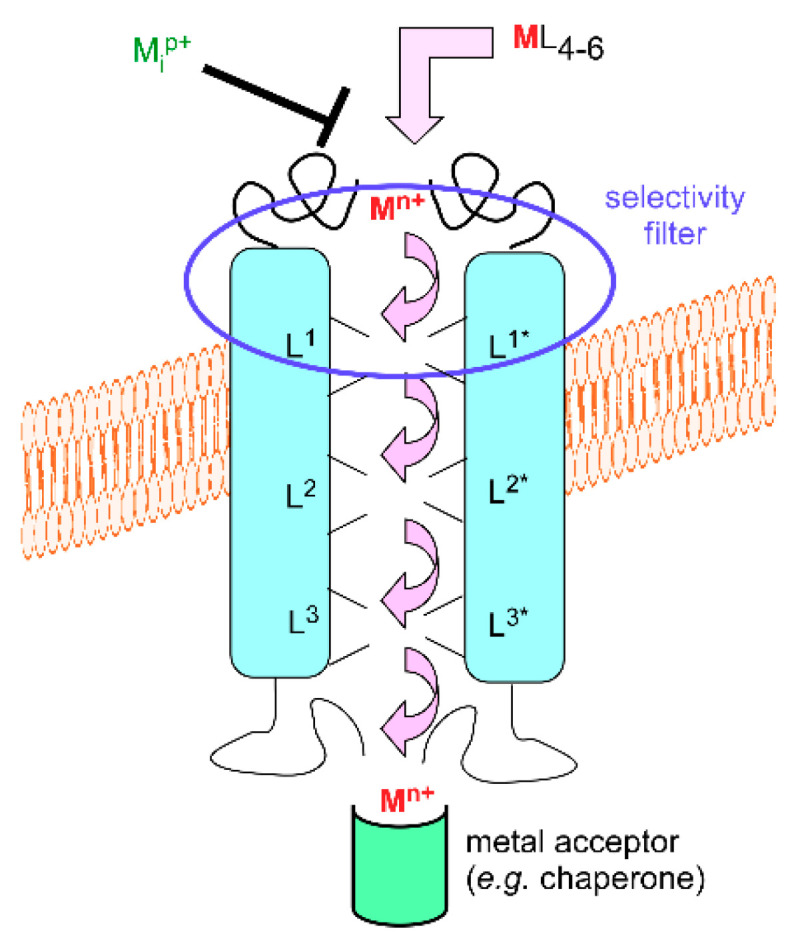
Schematic representation of a transmembrane metal transporter. M^n+^ (red) is the transported cation, L^1^, L^2^, etc. the transporter-associated transient metal ligand sets, and M_i_^p+^ (green) a potential metal inhibitor of transport.

Even though not all molecular details of copper uptake by CTR1 have been fully revealed, the mechanism sketched in the preceding paragraph (Figure 1) is widespread for a variety of metal cations, organisms or cellular types, locations, and transporter families. The source of energy for transport may vary (metal gradient, ATP, electrochemical gradient, etc.), but a general trend is that a chain of metal-binding sites leads the metal ion to its target. This implies that trans-membrane transporters of metals under their simple ionic forms are metal-binding proteins. However, they would be worthless if the metal ions to be transported stick to them. Hence, dynamic exchange and transient binding are mandatory to fulfill the transport function. To illustrate that dynamics supports the whole process, tight binding, as in the case of large concentrations of cadmium or lanthanide ions to calcium channels, modifies the transport function [16]. Furthermore, a few examples evidence that the transient metal-binding sites afford selectivity filters for metal cations. A single His_2_Asp_2_ site in the ZNT (SLC30A) family of mammalian zinc transporters excludes transport of the chemically similar cadmium ions [17]. The same general scheme of a succession of well-organized and thermodynamically and kinetically adjusted sites offers satisfactory rationales in a variety of situations. For example, polyanionic compounds, such as phytates, i.e., inositol phosphates, and polyphenols, bind essential metal cations, such as iron and zinc, in food. Their excess, as in nearly exclusively cereal-based diets, contributes to anemia and zinc deficiency by interfering with metal absorption. Two combined effects are at work here: decreased solubility of the diet-derived metal complex, hence decreased metal availability, and unsuitable transfer of the metal ions to the intestinal metal transporters. It may be safely stated in this respect that metal loading to the iron transporter Divalent Metal Transporter 1 (DMT1, SLC11A2) [18,19] and to the Zinc regulated transporter, Iron regulated transporter-like Protein (ZIP, SLC39A) family [20] is impaired when phytates, polyphenols, and other cation-scavenging compounds are present in the intestinal lumen. For this reason, food processing resulting in separating or degrading such cation-binding food molecules is useful to improve iron and zinc availability for animals [21].

### 3.2. Crossing Membranes Alone or as a Party?

In the microbial world, the critical fight for survival and growth depends on the availability of transition metal ions, which is supported by the synthesis or use of sidero- or zinco-phores for instance. In the case of iron, siderophores may be molecules of various sizes and complexity, such as citrate, desferrioxamine, or enterobactin, but all with suitable ferric iron liganding groups (carboxylates, hydroxamates, catecholates) [22]. Once the siderophore-iron complex interacts with the microbial cell, two strategies may be observed. One involves reduction of the complex at the cell surface and transport of the ferrous ion by transporters following the general picture described above for CTR1. The second requires more specific siderophore receptors that can recognize and transport the complex from the outside to the cytosol. There, mechanisms, such as siderophore degradation and complex reduction, help liberate the precious metal for internal use. Thus, in the first case, ferrous iron exchange occurs along the transmembrane transport, whereas the metal-binding site of the tight siderophore-iron complex changes only once inside the organism in the second case. However, in both cases, iron acquisition is a dynamic process at the metal-binding site that is coupled to even minute changes of the surrounding conditions, such as those triggering a redox transition or the distant modification, e.g., bond cleavage, of a ligand. The main objective of these different mechanisms is to decrease the affinity constant of the iron-bound species, hence dissociating the metal ion at the right place, at the right time, and in the suitable oxidation state for further use [23]. The possibility of transporting metals bound to organic molecules through membranes is clearly not restricted to the microbial uptake of iron bound to siderophores. Another example, among many, is provided by multidrug resistance proteins that contribute to expel divalent metal cations in the form of complexes with glutathione [24].

Taking the theme of metal exchange in the acquisition process one step further, endocytosis of the transferrin receptor is the main iron uptake system in mammalian cells. In this process, circulating transferrin binds one or two ferric ions loaded by oxidation of newly absorbed or recycled ferrous iron. Transferrin is recognized by its receptor at the surface of cells and internalized by clathrin-mediated endocytosis [25,26]. Decreasing pH in the endosomes triggers iron release from the complex, a member of the STEAP (six transmembrane epithelial antigen of the prostate) reductase family reduces the ferric ions, and the resulting divalent iron crosses the endosomal membrane via transporters, such as isoforms of DMT1. Therefore, here again, the molecular mechanism of the important uptake pathway involving an essential transition metal relies on a series of metal exchange steps involving precisely located and regulated proteins and enzymes.

## 4. Methodological Hurdles in the Study of the Dynamics of Intracellular Transition Metal Trafficking

One may be rightly impressed by the gathered knowledge over the last several decades about the diversity of ways transition metals cross membranes. However, once the metal reaches the interior of the cells, the current description of real-time sub-cellular metal trafficking is generally less clear. To follow the just presented example of transferrin endocytosis, the fate of the ferrous ions coming out from endosomes remains debated [27]. It may be posited that methodological problems impair such studies for a large part.

Indeed, although easier to say than to do, knowing how much transition metals are in or out of cells can be precisely obtained. Direct analytical methods, such as atomic absorption spectroscopy and mass or optical detection after formation of inductively coupled plasmas, continuously improve their sensitivity. They can be straightforwardly implemented because cells can be readily and rapidly separated from the medium. Radioactive isotopes have also been instrumental in measuring rates of transition metal transport across the plasma membrane. However, these methods are less conveniently implemented as soon as cells are broken to get access to the sub-cellular traffic; indeed, this requires more sample processing than simply separating cells, and more time, during which artifacts in the metal movements may happen.

### 4.1. Transition Metals Biophysics: Diverse and Sophisticated

The properties of transition metals make them nicely suitable to the application of biophysical methods that are of little or no relevance in other areas of biochemistry. The electronic structure of paramagnetic transition metal ions and clusters enables recording of electronic paramagnetic resonance (EPR) signals [28]. The usefulness of the method is witnessed by the detection of metals in complex biological systems as early as at the turn of the 1960s [29,30]. The characteristic properties of metals’ interactions with radiations afford ample application of X-ray absorption spectroscopy and derived methods (X-ray absorption near-edge structure (XANES) and extended X-ray absorption fine structure (EXAFS)). The contribution of species containing transition metals to near ultra-violet and visible absorption has supported informative resonance Raman spectra. On the nuclear side of the properties of transition metals, the Mössbauer effect of the iron atom has illuminated the study of iron proteins [31]. Even though the nuclear transitions of metals have not been largely studied by direct nuclear magnetic resonance (NMR) of metalloproteins, probably by lack of convenient probes and implementation, the influence of the peculiar electronic structure of transition metals on the surrounding ‘organic’ nuclei has received widespread attention. This is true, for instance, for magnetic resonance imaging: the presence of paramagnetic transition metals perturbs the relaxation properties of nearby protons or other nuclei, which enables the detection of endogenous metal deposits, in neurodegenerative diseases in particular [32]. Additionally, exogenous metal labels have found a range of applications in NMR following the same principles. Solid-state NMR has more recently developed the usefulness of the method in the study of metalloproteins [33].

### 4.2. Transition Metals Biophysics: Necessarily Limited

Thus, all these methods have been instrumental in characterizing the metal site(s) of metal-binding proteins, in complement to structural methods. However, purified or otherwise processed proteins to obey the methodological requirements are not exactly in the conditions found inside cells. The main drawback is the loss or the alteration of the dynamic component of these proteins’ behavior that is inevitably modified in pure diluted or very concentrated solutions, or in the solid or frozen state. Further, the sensitivity of each of these methods is limited, although improvements have been tremendous for seemingly all of these methods over the years; consequently, the concentration of samples must often be increased in order to reach sufficient signal/noise ratios. In addition, the temporal resolution of most of these methods does not correspond to the kinetic steps involving metal exchange that one would like to monitor. An exception to this statement may be NMR, the temporal resolution of which can be adjusted by the strength of the applied magnetic field available for series of instruments. NMR can record molecular movements, such as protein conformational changes or large variations of the magnetic properties of the sample, hence indirectly binding or release of metals. However, being able to reconstitute in real time the sequence of events involving the movements of metals inside cells between several partners and in a changing environment is a formidable task that cannot yet be straightforwardly achieved. Not surprisingly excellent approaches aiming at this purpose involve the combination of several biophysical methods [34].

## 5. Labeling Transition Metals in Cells: Pros and Cons

### 5.1. The Resolution Problem

Thus, for the moment, following the intra-cellular trafficking of transition metals and their binding molecules is complex, requires time, and remains an exceptional feat. Mapping the distribution of heavy elements, including metals, in combination with other analytical methods inside cells has become available and with ever increasing resolution, at synchrotron facilities in particular [35]. However, samples have to be processed and immobilized for this purpose. One way of overcoming this static-only view of the problem would be to use fluorescent molecules binding specifically to the metal of interest and monitoring the fluorescent signal in real time in live cells. This approach has provided invaluable tools in the study of the roles of calcium [36] and zinc [37] in Biology. However, the divalent Ca^2+^ and Zn^2+^ do not readily exchange electrons, which does not interfere with increased (turn-on) fluorescence upon binding. In contrast, the easy uptake or release of electrons by most transition metal ions (those with unfilled *d* orbitals) turns them into fluorescence quenchers, meaning that metal binding to the probe results in a less convenient negative (turn-off) fluorescent signal. Some progress has been made to circumvent this problem [38], but applicability remains tedious in most cases.

In an ideal world, integrating the above fluorescent methods in super-resolution fluorescence microscopy [39] would come close to precisely monitor intra-cellular metal exchange. Unfortunately, even though “super-resolution” means that the diffraction barrier of optical microscopy is overcome, the high-resolution microscopic technics are still far from picturing the changes in the coordination sphere of a metal. In addition, the time needed to acquire images, despite continuous improvements, is longer than most molecular events occurring near the metal.

### 5.2. The Specificity Problem

Beyond the above-indicated limitations, other potential difficulties in monitoring metals inside cells have to be considered with appropriate controls. They include the ability to master the localization and concentration of the metal probes, their metal specificity inside cells that may be challenged as compared to validation in vitro, and the always-questionable introduction of engineered molecules to observe the endogenous object of interest. Indeed, small molecules binding metals do trap them. They divert them from their normal path. They usually perform best on loosely bound complexes, and, as exogenous species, may perturb the integrity of cells, at least above concentrations that may be needed to get sufficient signals. These drawbacks may be amplified with labeled proteins that influence the kinetics of metal-centered reactions and associated cellular events, due to their size and their larger tumbling time as compared to those of short-lived endogenous metal ligands. Last, metal specificity is a constant effort with all designed metal probes, and it is difficult to achieve given the largely shared coordination properties of subsets of transition metals. In addition, the biological interest in transition metals very often lies in their ability to switch oxidation states. Thus, the problem is worsened by the need to monitor the same element with different electronic configurations. The binding properties, including of probes, are strongly modified by the redox change, which affects specificity. The preceding few lines should show that applying the intrinsically integrative high-resolution microscopies to metals in biology or metal-binding proteins is far from being a mastered approach.

### 5.3. Monitoring Labeled Metals and Proteins: Work in Progress

However, the just recalled impediments in following fluorescently labeled metals or metal-binding proteins should not dismiss the various successes obtained with the derived methods in the study of transition metals in Biology. An example is given by monitoring iron uptake and use in mammalian cells via the transferrin-transferrin receptor system. Use of radioactive isotopes, fluorescent labeling of transferrin, and sub-cellular fluorescent makers, all followed by appropriate detection methods, including confocal microscopy, led to the proposal that internalized iron was directly injected from endosomes into the main organelle for biosynthesis of iron-proteins, namely mitochondria [40,41]. Further developments along the same lines supported the hypothesis of “tunneling” iron to ferrochelatase for incorporation into hemoglobin in highly iron-demanding reticulocytes [42]. Yet, the still limited spatial and temporal resolution of this combination of methods leaves room for questioning the universality of this phenomenon [43]. Clearly, more work integrating qualitative [44] and quantitative [45] data is needed to clarify the traffic of iron in and out of the cytosol of mammalian cells.

By taking proper account of the present biophysical frontier in the study of metalloproteins, getting close to actually seeing transition metal traffic in intra-cellular space and time is a stimulating challenge that will undoubtedly keep talented scientists busy for many years to come.

## 6. Divide and (Try to) Conquer in Monitoring Transition Metals

The above presented approaches aiming at closely monitoring transition metals in minimally processed biological samples are relatively recent developments. In a previous period, the biochemistry of transition metals has largely benefited from the huge progress of separation and characterization methods since the middle of the 20th century. Metal-oriented biophysical techniques have been recalled above, but it should be kept in mind that their implementation was backed by less specific biochemical methods affording scientists tools for focused investigations.

Indeed, the increasing availability of purified metalloproteins over the years revealed a wealth of new information. From the mid-1980s onward, microbial genetic engineering allowed biochemists to produce proteins in abundance and to change their sequence at will. These developments opened a wide range of new investigations and consequent discoveries. Metal-binding proteins were not exceptions in this respect.

In the following, the aim is not to shed a negative light on all the data produced by the reductionist approach and structure–function relationships: they form the base of a great deal of knowledge on metal-binding proteins, and this review would fall short of giving credit to all accomplishments. Rather, the purpose is to highlight frequently overlooked, and difficult to publish, pitfalls associated with biochemical metal exchange that newcomers, and maybe others, may experience in this field.

### 6.1. Metal Exchange as a Probe

The ability of metals to exchange at the active site of enzymes was early used as a means to probe the structural and mechanistic properties of ill-characterized metalloproteins at the time. For instance, the evidence for association of zinc with proteins dates back to the 1940s [46], and substitution of zinc for cobalt in purified proteins gave useful insight into the molecular details of numerous zinc-enzymes by highlighting features hidden by the often spectroscopically silent zinc [47,48]. Such metal substitutions were guided by the purposes of the investigators to get proteins with their full load of the wished transition metal: this approach was successful in most reported examples.

### 6.2. The Metal Trap of Model Organisms

**Figure 2:**
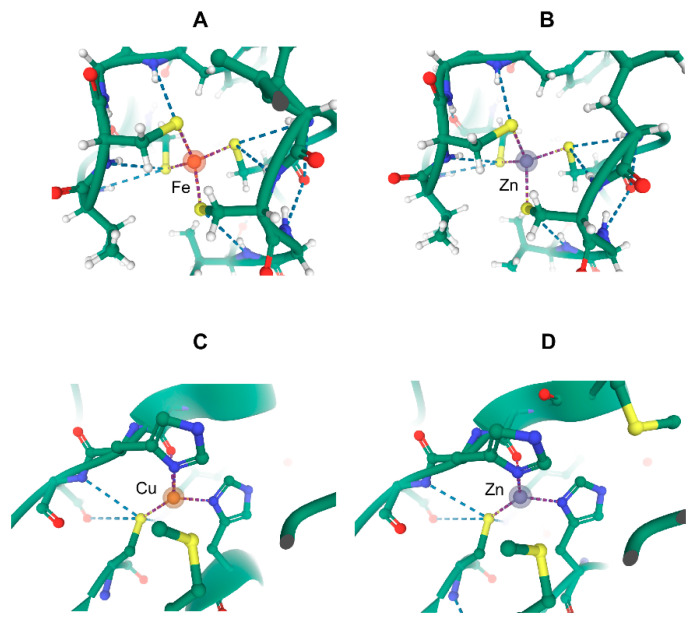
Comparison of the metal-substituted recombinant *Clostridium pasteurianum* rubredoxin and *Pseudomonas aeruginosa* azurin produced in *Escherichia coli*. The panels show the metal ion coordination spheres for (**A**) Fe(III) rubredoxin (RCSB PDB 1IRO, resolution 1.10 Å), (**B**) Zn(II) rubredoxin (1IRN, resolution 1.20 Å), (**C**) Cu(II) azurin (5AZU, resolution 1.90 Å), and (**D**) Zn(II) azurin (1E67, resolution 2.14 Å). The images were generated on the rcsb.org site using Mol* ((**D**). Sehnal, A.S. Rose, J. Kovca, S.K. Burley, S. Velankar (2018) Mol*: Towards a common library and tools for web molecular graphics MolVA/EuroVis Proceedings. doi:10.2312/molva.20181103).

However, the advent of genetic methods allowing researchers to obtain large quantities of otherwise difficult-to-purify proteins were the ground for metal exchange phenomena at the metal-binding site of metalloproteins, often in an insidious way. Model microorganisms, such as *Escherichia coli* or *Saccharomyces cerevisiae*, were the workhorses of recombinant technologies. The availability of various mutants and the ease of manipulation of their genetic material from the late 1980s onward provided tremendous power to the thorough structural and mechanistic characterization of a variety of proteins, including metallo-ones. However, producing proteins in abundance in given non-native hosts led to unexpected metals at the binding site(s) of recombinant proteins. For instance, rubredoxins are small proteins initially characterized as iron binding through four cysteine residues in anaerobic bacteria [49]. They can be readily produced in *E. coli* as the iron-containing protein [50]. However, upon characterizing this recombinant material, it soon appeared that another form was present in *E. coli* lysates, namely the zinc-substituted protein in which zinc is quasi-isostructural to iron [51,52] (Figure 2A,B). Interestingly and concomitantly, a similar observation was made with recombinant *Pseudomonas aeruginosa* azurin [53] (Figure 2C,D). This type I copper protein and rubredoxin are parts of electron transfer chains. Beforehand, exchange at the electron-transferring sites with a diversity of non-native, including redox inactive, metals had been demonstrated for both purified proteins. Among the different metal-substituted forms, the ones with the native metal were found to be more stable than the zinc-containing ones [51,54]. *Escherichia coli*, the microbial host used in the mentioned series of studies, can be grown in minimal media with perfectly controlled metal concentrations. By varying the relative concentrations of iron and zinc in such a medium, the relative amounts of recombinant iron- and zinc-rubredoxin accurately reproduced the metal distribution of the medium (Jacques Meyer, personal communication). It follows that *E. coli*, which does not hold any rubredoxin gene in its genome, is unable to discriminate between iron and zinc at the metal-binding site of *Clostridium pasteurianum* rubredoxin. Of note, the implemented gene expression system [55] stops any transcription at the time of induction except that of the gene of interest. Thus, assembly of the exogenous rubredoxin exclusively occurs with the *E. coli* biosynthetic machinery already present before induction, without means to adjust to the new biosynthetic needs. It must be concluded that *E. coli* cannot quantitatively incorporate the right metal in a protein as simple as rubredoxin, which has no other high-affinity binding site than that formed with the four cysteine residues [49]. Another conclusion from these observations is that, in contrast to *E. coli*, the rubredoxin natural host *C. pasteurianum* does exclusively address a single metal at the active site of this protein following a still uncharacterized pathway. Indeed, the presence of any other rubredoxin form besides the iron-containing one in *C. pasteurianum* is unlikely to have escaped detection since iron and zinc rubredoxins co-purify and the absorption spectra of the mixture easily indicates whether iron rubredoxin is exclusively present (J-M.M. and J. Meyer, unpublished observations). The zinc- and iron-rubredoxins can only be separated by high-resolution anion exchange chromatography [51].

### 6.3. Serendipitous and Understated Metal Binding

**Figure 3:**
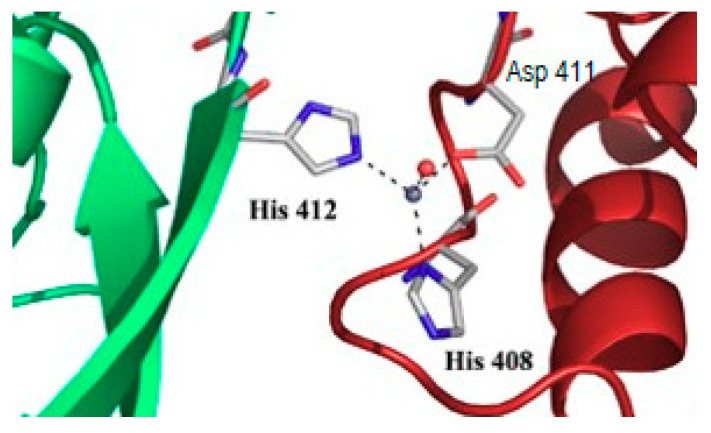
Interfacial zinc binding to Iron Regulatory Protein 1. Zinc was found associated with the protein in the C222_1_ crystals [3]. The zinc ion interacts with one histidine (number 412) from one polypeptide (green), and one histidine (408) and one aspartate (411) from another polypeptide (red) in the crystal system. A water molecule completes tetrahedral coordination. RCSB databank entry: 2B3X.

In the introduction, the occurrence of unexpected metal binding to proteins in structural studies was recalled, e.g., [3,4]. The presence of a supposedly alien metal ion (Figure 3) may reflect the composition of the solutions used in the crystallization process, but such ions may also be selected by the protein without any intentional addition in the medium [3]. Thus, binding of transition metals to proteins may remain undetected, unless metal-specific methods, such as X-ray fluorescence, are applied to the sample, sometimes for other purposes than detecting any cryptic metal. This apparent difficulty has been profitably used to solve many crystallographic protein structures by expediting the phase problem [56]. The presence of metal-binding sites at the surface of proteins (Figure 3) contributes to crystal packing in cytosolic aconitase [3], and it might be an indication that the interaction also occurs in other states than crystals. In the case of the different forms of cytosolic aconitase/Iron Regulatory Protein 1, evidence for the interaction of zinc, cadmium, and trivalent metal ions was obtained in solution with consequences on the properties of the protein, including solubility, hence activity [57]. These observations were made with purified proteins, and their relevance inside cells is usually difficult to demonstrate.

The traps in which scientists with interest in metalloproteins may fall using recombinant proteins go beyond the above metal exchanges or occasional binding. They also include artificial metal binding to proteins that do not require them for function.

### 6.4. When Metals Spoil the Show

A popular strategy is to produce recombinant proteins with histidine tags to expedite purification. Yet, the production of proteins, particularly those encoded by the genome of high eukaryotes, occurs under different conditions in the cytosol of *E. coli* than in their native hosts. In particular, such proteins do not correctly fold without the proper eukaryotic quality control systems and they are prone to forming inclusion bodies. In addition, those containing cysteine-rich domains may grab metals available in the bacterial environment and trap them. For instance, the mammalian protein onzin (PLAC8) is cysteine-rich: the mouse sequence contains 15 out of 112 aminoacids, i.e., more than 13%, a value similar for other mammalian species, whereas the average cysteine frequency in the proteomes of vertebrates in general is of the order of 3.5%. Mouse onzin was found in inclusion bodies when produced as a fusion with glutathione-*S*-transferase in *E. coli*. When iron was added to the medium during production, the inclusion bodies became dark (Magali Chemali, Jérôme Garin, personal communication), and they released the characteristic smell of hydrogen sulfide when treated with hydrochloric acid (J-MM unpublished). These observations strongly suggested the presence of iron-sulfur clusters. However, when 32D mouse cells were labeled with radioactive iron-loaded transferrin, anti-onzin antibodies did not fix more iron than the pre-immune serum used to raise these antibodies (J-MM unpublished). Therefore, onzin likely does not bind iron in mammalian cells. Its possible involvement in iron homeostasis and biogenesis of iron-sulfur clusters, under some conditions at least [58], may be mediated by its interaction with components of these pathways rather than direct binding of the metal. Furthermore, the opportunistic binding of metals by cysteine-rich domains in heterologous proteins is not restricted to iron-sulfur clusters, as zinc [59] or other divalent metals [60] may also be found associated with such proteins.

### 6.5. Questioning Metal ‘Reconstitution’

If a reason exists to suspect that some proteins interact with metals and if they are nevertheless synthesized as recombinant apo-proteins, it may be tempting to ‘reconstitute’ them by adding the supposedly ‘natural’ metal ions after purification. For instance, hepcidin has become the master regulator of metazoan iron homeostasis over the last two decades. Its mature form recognizes its target, the iron cellular exporter ferroportin, mainly by its 5 N-terminal amino acids [61], and it has 8 cysteines among the 20 amino acids of its C-terminal sequence. It is thus not surprising that metals including ferric ions bind to the reduced peptide in vitro [62], but the physiological relevance of such binding has never been demonstrated and it is fairly unlikely for a secreted protein folding via formation of disulfide bridges [63]. Indeed, heterologous production of mature hepcidin in engineered *E. coli* with proximal disulfide bridge-building ability via a thioredoxin domain afforded the properly folded oxidized active hepcidin without evidence of transition metal binding [64]. Similarly, production of C-terminal histidine-tagged hepcidin in the yeast *Pischia pastoris* was not reported to be associated with transition metal binding [65], even though the risk of anecdotal metal binding is amplified when His tags remain associated with proteins after synthesis. For example, in vitro reconstitution of N-terminal His-tagged Iron Regulatory Protein 1 with iron and sulfide yielded a protein with a linear cluster, the aconitase activity of which was not reported [66], and which was previously found in the denaturated purified mitochondrial aconitase enzyme [67].

**Figure 4:**
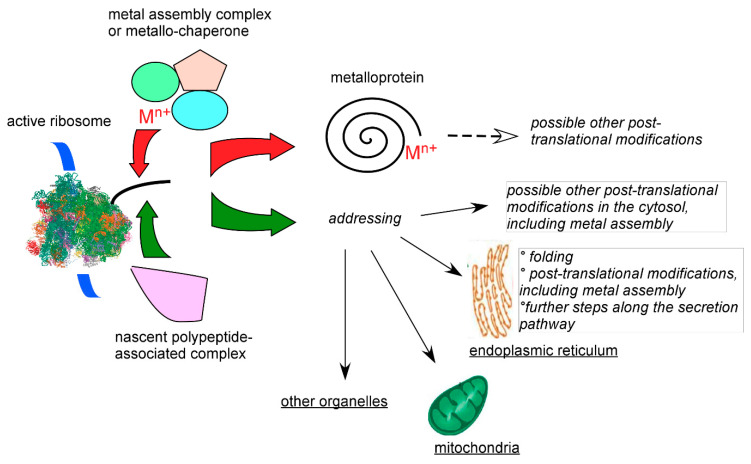
Schematic competition between metal insertion and other post-translational modifications on newly synthesized proteins bearing metal ligands. On the left, the blue ribbon represents the translated mRNA and the black tube the produced protein on active ribosomes. Cytosolic metal insertion depends not only on the recipient protein but also on the cellular environment. What commits the newly synthesized polypeptide either to (top, red arrow) swift metal insertion, or (bottom, green arrow) to further cytosolic processing or translocation to organelles in eukaryotes with delayed metal assembly is unknown. Metals insertion may indeed occur in organelles as better known in mitochondria for iron-sulfur or hemoproteins.

From the preceding paragraphs, it appears that a range of activities and conditions that cannot yet be introduced in vitro modulates the combinatorial interactions between transition metal cations and cysteine-rich proteins. In bacteria, such as the Gram (-) *E. coli* largely used for heterologous protein production (*vide supra*), protein disulfide isomerases are periplasmic enzymes involved in the oxidative folding pathway, and the ribosomal cytoplasmic environment is mainly reducing in the absence of stress. Therefore, metal delivery systems probably have time to act on nascent proteins before disulfide bridges are formed. In eukaryotes, quality control and addressing mechanisms are coordinated and act on active ribosomes to avoid waste of cellular resources and potential toxic effects of deficient translation and translocation [68,69]. How metalloproteins balance the function of these complex and successive monitoring systems with incorporation of metals or metal prosthetic groups is presently a fully open question. However, in the case of secreted, cysteine-rich, proteins, such as the above mentioned hepcidin, translocation to the endoplasmic reticulum and further processing and quality control down to the export vesicles may shield the newly synthesized proteins from irrelevant metal-binding machineries. These pathways are illustrated in Figure 4. Following this simple reasoning, deficiency in these tight monitoring systems may lead to improper and deleterious metal–protein interactions, as observed in a variety of diseases, such as Alzheimer disease, spongiform encephalopathy (mad cow disease), Parkinson disease, and other tauopathies, with aberrant metalation and oligomerization of the tau protein, prion protein (itself a copper protein), and α-synuclein, respectively.

## 7. Cellular Targeting of Transition Metals

### 7.1. Different Metal Solutions to the Same Problem

In view of the multiple interactions metalloproteins may display with transition metal ions, as exemplified above, a question mark soon appeared as to whether the metals found in purified proteins, usually at the active site, actually reflected the situation in the native environment of whole cells. Indeed, some proteins and enzymes from different organisms, particularly bacteria, were characterized with variable identities and concentrations of different metals [70]. This is particularly, but not only, true for metals, such as ferrous and manganous ions, that are close in the series of divalent metal-complex stabilities [71,72]. A case in point is the β2 subunit of class I ribonucleotide reductase (RNR) [73]. This metallosubunit of the enzyme stabilizes a radical that plays a mandatory role in the reduction of nucleotides. Surprisingly, the dinuclear metal center of subunit β2 may hold different metals in the form of di-ferric (e.g., class Ia) or di-manganic (e.g., class Ib) active sites. Therefore, a given activity may not be uniformly associated with a site holding a specific metal. This can be true in a single organism depending on the conditions, sometimes by use of different isomers, [74], or by comparing different organisms, such as those employing class Ia and class Ib RNR.

### 7.2. Several Metal Problems with the Same Protein or Enzyme: Is Toxicity by Metal Replacement So Much Relevant?

Reciprocally, the inclusion of a different metal than the original one in a metallo-enzyme or protein may affect the activity. This phenomenon has been proposed as a mechanism of toxicity although its importance should not be overstated as already warned before [75]. For instance, cobalt salts have long been known to mimic hypoxia when applied at relatively high concentrations, generally several tens of µM on cell cultures. Most often, replacement of the active site iron by the inactive Co^2+^ at the active site of hydroxylases that modify the hypoxia-inducible factors α is put forward, but various lines of evidence do not support this proposal. First, the supposed cobalt-containing prolyl- or asparaginyl-hydroxylases have not been characterized with unambiguous proof they do form in cell cultures. In this respect, it may be noticed that the active site of these enzymes displays some flexibility, enabling binding of several metal ions [76]. Second, the transcriptional effects of cobalt salts, on the one hand, and iron chelators, on the other hand, do not exactly overlap, and their respective inhibition of the different hydroxylases is not the same in vitro and inside cells [77]. This contrasts with the large sensitivity of prolyl- or asparaginyl-hydroxylases to iron depletion [78,79], which should favor the replacement of iron by cobalt or other divalent transition metals in Fe^2+^-2-oxoglutarate-dependent dioxygenases [80,81]. Third, the absence of cobalt, not bound to vitamin B_12_, in mammalian cells turns it into a toxic element able to negatively interact with a variety of biomolecules. The mechanisms of cobalt induction of hypoxia have been recently examined [82]. This example, as well as many others that should become as well documented as this one, emphasizes again that metal replacement on metalloproteins should not be hastily proposed to explain metal toxicity. Instead, the field of the mechanisms of toxicity triggered by non-essential metals offers a wide and stimulating range of chemical and biological investigations that will certainly develop in the future.

### 7.3. Chaperone, Channeling, and Active Site Synthesis of Metalloproteins

The difficulties outlined above illustrate the problem for scientists and cells alike to put the correct metal in a metalloprotein. Indeed, it is now very clear that the primary and even ternary or quaternary structures of a protein may be necessary, but certainly not sufficient, for assembly of the proper metal or metal group at its active site as it was initially thought. The changing view of the involvement of accessory agents to carry out this task arose from the strong development of genetic studies, including improved sequencing methods, and characterization of proteomes. A typical example is that of iron-sulfur proteins. Indeed, this very large family of proteins bind the metal centers with a majority of cysteine residues, some of them organized in easily recognized sequence signatures. Many of them can assemble clusters when provided with the suitable reactants in vitro. However, the most complex of these centers, such as those found in the catalytic subunit of the nitrogenases of diazotrophs, cannot be ‘reconstituted’ from simple reactants in the apo-subunit. Even the most common forms of iron-sulfur clusters interconvert [83], which shows that a given protein may accommodate different cluster types, and this has functional application in cellular sensors [84]. Bacterial genomes revealed operons involved in the biosynthesis or the repair of iron-sulfur centers. Homologs of most genes identified in bacteria were later found in eukaryotes, where additional proteins participate in the biosynthesis of these clusters to deal with sub-cellular localization and target specificity. Thus, proper assembly of iron-sulfur clusters in the proteins designed to hold them requires a series of protein–protein interactions, with some participants bringing cluster fragments and reactants to the complex [85,86,87]. Some participants in metalloprotein assembly pathways shuttle the transition metal to its targets and received the name of chaperones. The earliest identification of chaperones was for copper [88,89], a relatively rare yet essential transition metal that is redox active. Cells cannot afford to waste such a precious material, but they cannot let it redox cycle and catalyze unwanted oxidative reactions, hence the crucial role of copper chaperones. It is likely that chaperone molecules exist for all biologically essential transition metals, although many remain to be discovered.

### 7.4. Tuning Transition Metals’ Reactivity in Metalloproteins

The high reactivity of transition metals is a biochemical asset but a threat as well. The range of functions in which metalloproteins participate is very large. As well as putting the right metal in the right place requires costly means, the need to properly orient the reactions or interactions in which a metalloprotein is involved is not a simple cellular problem. This is probably why cells have developed complex molecules to which metals bind as a way of preconditioning their reactivity. This way, prosthetic groups leave only a fraction of the ligand positions available on the metal at the active site, which limits the number and kind of reactions that may take place. Among prosthetic groups, the most widespread are those using a tetrapyrrole ring that only leaves the two axial positions of iron or other transition metals (Ni in F430 of methanogenic archaea) for ligand exchange. Other examples include cobalamin (vitamin B_12_), of which animal cells have lost the biosynthetic pathway, and molybdopterin. The biosynthesis of protoporphyrin IX involves successive reactions that are located partly in and partly out of mitochondria, and the ferrous ion needed to form a heme is incorporated as the last step by the enzyme ferrochelatase, itself bearing an iron-sulfur cluster in animal cells [90]. Thus, large cellular resources are devoted to the synthesis of complex prosthetic groups, emphasizing the crucial need of properly reacting transition metals for all forms of life. One of the most recent examples of the importance of this pathway relates to heme oxygenase activity, which degrades heme, in the mechanism of damage occurring during the new and presently widespread SARS-CoV-2 infection [91]. Despite the constrained coordination sphere built by the prosthetic group, metal exchange does also occur in metalloproteins binding them. For instance, under iron deficiency zinc is incorporated, probably by mere, non-catalyzed, binding, into protoporphyrin IX, and the latter complex can be used as a biomarker for anemia and in a large series of chronic diseases in which inflammation is enhanced [92], the COVID-19 disease that prevails at the time of the present writing not being an exception.

## 8. Extreme Sensitivity of Transition Metal Exchange Reactions to Cellular Conditions

When compared to conventional proteins that do not, or marginally, require metals, the occurrence of complex pathways leading the right metal or metal group to its target(s) is likely due to the absence of any quality control system for metalloproteins. Indeed, improperly folded proteins are readily recognized, labeled, and directed to degradative pathways, such as the proteasome, e.g., [93]. No such process designed to check the correct incorporation of a transition metal at the active site of a metalloprotein is known. By far, not all apo-proteins fold differently from their holo-counterpart, especially the largest ones. Thus, cells seemingly have no way to get rid of useless apo-proteins, all the more so as the protein without metal may have its own function: an example is metazoan Iron Regulatory Protein 1 that interacts with its RNA partner, the Iron Responsive Element, in the absence of bound metal, in this case a cluster [94]. Even when apo-proteins fold around a metal, cells have no way to selectively identify and eliminate metalloproteins holding the wrong metal.

### 8.1. Coordinating Metal Assembly and Metalloprotein Folding: A Key Component of Stability

The tight connection between protein folding and metal incorporation is an important contributor to the correct synthesis of metalloproteins. Indeed, the tridimensional structure of metalloproteins, or at least of some of their domains or subunits, depends on the presence of the metal or metal-associated group, e.g., [95]. This is one of the reasons why cells have developed metal incorporation pathways, not only for selectivity of metal addressing but also for help in the folding pathway. For instance, among the accessory proteins involved in the biosynthesis of metal sites, several ensure the correct folding of the protein to avoid aggregation but also to prepare the active site for the reception of the metal. A particularly detailed example is provided by the biosynthesis of iron-sulfur proteins. At least the products of the mammalian genes *ISCU*, *HSC20*, *HSPA9*, and *NUBP1*, among around 30 involved in biogenesis of iron-sulfur clusters, appear to play such a role of chaperoning the assembly complex on the correct recipients in different sub-cellular locations ([96,97,98,99] for recent reviews).

A difference should be made between metal binding to nascent peptides/proteins with some metal contribution to folding, and metal exchange in fully assembled proteins. The former is a process that should generally poise the protein in a stable state to secure lasting function. Other post-translational reactions, such as the formation of disulfide bridges, contribute to folding, stability, and function [100]. Metal exchange on folded proteins is more likely to occur when stability is compromised as for partly disordered, including small, proteins, or when environmental conditions lead to unfolding. For instance, it was soon shown that iron-sulfur clusters readily exchange with externally provided cluster components in chaotropic agents ([101] and Table 1). Interestingly, archaea and bacteria sometimes have a backup system for biosynthesis of iron-sulfur clusters encoded by the *Suf* operon in parallel with the *Isc* one [102]. Key products of the *Suf* operon have been shown to be more stable than corresponding ones of the *Isc* operon and to participate in cluster building or repair under stress conditions. Interestingly, this kind of backup system seems to have been replaced in vertebrates by other mechanisms, such as cluster repair carried out by the mitoNEET protein of the outer mitochondrial membrane [103]. This protein appears to monitor aspects of iron homeostasis as they relate to dysfunction of oxygen use through generation of reactive oxygen species, and other redox imbalance reactions in which metals are key participants.

In all the above examples centered on iron-sulfur proteins, and in many additional ones, reactions that can be formally considered as metal exchange are central to the correct delivery of metals to active sites, the repair of these sites in case of insult, and recycling or disposition of metals in case of denaturation.

### 8.2. Transition Metals Turned Wild: A Major Cellular Threat

Probably the most studied, and invoked, insults that cells may experience are oxidative stress or its equivalent with nitrogen derivatives, namely conditions increasing the formation of so-called oxygen or nitrogen reactive species. Among these ‘reactive species’ are small molecules, such as hydrogen peroxide, the hydroxyl radical, or peroxynitrite, that are not equally reactive with different molecules but that may modify numerous cellular components. They are usually associated with cellular damage when their concentration is high enough. Some of them also play signaling roles by shifting the intracellular redox equilibrium and, sometimes, specifically reacting with selected cellular components acting as signaling relays. The dynamics of transition metals’ reactivity strongly impinges on the emergence or development of oxidative or nitrosative stress. Indeed, the reactivity of unshielded or partially shielded forms of iron or copper inside cells catalyzes the formation of the most reactive species, as in the case of ferroptosis for example [104]. To survive, cells have to damper the insult, and they often do so by acting on the homeostasis of transition metals. To take a single example, the Iron Regulatory Proteins are cellular regulators of iron homeostasis that react with, and whose activities are sensitive to, reactive oxygen and nitrogen species [105,106,107,108,109]. Recovery or enhancement of the regulatory activities has been shown to require the involvement of small electron shuttles, such as thioredoxin [110], that are now known to activate by release of their bound iron-sulfur cluster [111] in addition to reduction by thioredoxin reductase. Thus, the inter-connection between homeostasis of transition metals, here iron, and redox homeostasis goes both ways: the former catalyzes the establishment or imbalance of the latter, but the latter contributes to maintain the former. The thorough characterization of the quantitative traits determining this inter-connection will certainly be an area of continued interest in the years to come because of its mechanistic importance in a very wide range of conditions, including many pathological ones.

It has just been indicated that external conditions, such as a redox shift, determine the ability of proteins to hold metal or metal prosthetic groups. The dynamics of metal exchange, such as alterations of mitoNEET proteins associated with many diseases (e.g., [112]), underlies a very large array of pathologies that cannot be extensively listed here. However, a very important example is the competitive binding of metals to the products of the respective genomes is a key aspect of the pathogen–host interaction in the immune response [113,114]. The rather unselective metal-binding protein calprotectin is instrumental in starving the pathogen for transition metals and preserving the metal-based activities of the host, including those needed to face invasion [114,115]. It is thus not surprising that calprotectin appears as a severity factor in the SARS-CoV-2 infection [116].

## 9. Puzzling Interactions of Transition Metals and Metal-Binding Molecules with Proteins

The versatility of the interactions involving metalloproteins also transpire in the observation of unexpected ligands binding to them. This may occur with metallo-drugs that deliver their non-essential/toxic metal or exchange it at the active site of metalloproteins [117,118]. Interestingly, drugs and other molecules not only bind to the active site, but they may also participate in oligomerization or bind to other sites to affect function [118]. In the quoted example of platinum-based compounds binding to the copper chaperone ATOX1, it appears likely that the resistance mechanism to these anticancer drugs involves drug delivery by loaded ATOX1 to the copper export proteins ATP7A and ATP7B expelling platinum. This way, platinum is diverted from its intended intracellular targets, DNA and sulfhydryl-rich molecules, thus failing to inhibit DNA replication and transcription.

Taking the versatility of metalloproteins one step further, metal or metal prosthetic group binding to some proteins, although clearly demonstrated, may have questionable physiological relevance in some cases. For instance, the prosthetic group heme is both a very hydrophobic and reactive molecule. Its normal fate is to bind to coordinately synthesized apoproteins to form hemo-proteins and enzymes. Yet, all cellular reactions of unbound or loosely bound heme remain to be comprehensively established, although regulation of the transcriptional repressor Btb and Cnc homology 1 (Bach1) and eukaryotic initiation factor-2α kinase (HRI) has been extensively documented [119,120]. Heme binds to many different proteins [120], and even though some of these proteins can be unambiguously associated with heme synthesis, transport, or degradation, the role of others is enigmatic. For instance, heme-binding protein 1 (HeBP1) was discovered through its induction in the highly demanding pathway of hemoglobin synthesis [121], and, as the name says, it does bind heme, protoporphyrin IX, and other tetrapyrroles [122,123,124]. However, the roles of HeBP1, of its ortholog SOUL (HeBP2), and their domains and fragments now go beyond direct heme metabolism, with involvement in cell death mechanisms and the immune response in metazoans. Such functions may be due to late developments during evolution that have little connection to iron and heme homeostasis [125]. Even though neurons of neurodegenerative animal models and Alzheimer’s disease patients overproduce HeBP1 and are specifically more sensitive than controls to hemin-induced cell death among other death inducers [126], the actual role of heme binding to HeBP1 in the surprising enhancement of neuronal cell death and in other cellular events remains to be delineated [127]. The example of the HeBP1/SOUL family of proteins deals with binding of a prosthetic group in which the metal may not be the main contributor to affinity, but it exemplifies the versatility of proteins classified as metalloproteins in their interactions with metals and their functions. The possible activities of metal-devoid metalloproteins are known in only a few cases, and it is a sub-domain of the increasingly important one of the moonlighting proteome that will likely become more scrutinized in the forthcoming years.

## 10. What May Be the Molecular Basis of Specificity for Cellular Transition Metals?

It should appear from the preceding paragraphs that, whereas transition metals must be available and their intracellular protein hosts must be in a state enabling correct folding around them, these two parameters fall short of explaining all traffic, molecular recognition, and exchange reactions involving metals in biological contexts. Furthermore, the cellular whereabouts of transition metals in animals have to fulfill the needs of both the relatively stable situation encountered by mature and specialized cells, and the moving and transient biochemical landscape of cells in developing organs, harmful environments, and other changing conditions. Clearly, the biosynthetic activities and the required concentrations of transition metals cannot be the same in all situations.

These issues make the question of defining the basis of cellular specificity for a given transition metal a very difficult task. Recently, the interplay between metal availability and free energy of binding in a series of bacterial metal sensors has been considered [128], and prevailing and insightful ideas in this area have been summarized [129]. The key combination appears to be that of *i*) a suitable metal concentration, *ii*) its availability in a chemical form enabling straightened and expedite delivery, and *iii*) a tuned affinity of the protein host. In the more complex situation involving organelles in eukaryotes, the intracellular trafficking and distribution of metals should contribute to this combination: this is in line with the notion of muffling introduced some time ago to explain modulation of cellular metal buffering in mammalian cells [130], following application to the amplitude of pH shifts [131].

None of the above interpretations and conclusions could have been reached without specifically designed experimental setups and analytical methods, and accurate and extensive quantitative data. Attempts at any comprehensive understanding of metal homeostasis cannot avoid such a depth of analysis as witnessed by studies in the minimally autonomous, i.e., able to grow in isolation on a single substrate, and model eukaryote *Saccharomyces cerevisiae* [132,133,134]. However, integration of the data available for this supposedly simple organism requires more than 150 variables and tens of reactions influencing them [135,136]. Whereas the latter modeling effort represents a formidable achievement, the data on which it is based suffer from the kinetic imitations indicated above, relative to the quickest steps of iron exchange and their potential effects on transient iron homeostasis. Explicitly taking fast kinetics into account would translate into more components/variables, more reactions, and more parameters to be evaluated in order to implement more integrative models. The just referenced work [135,136] certainly provides major insight, but it is easy to realize how more complex the situation will become when shifting away from a near steady-state condition as for growing yeast on a single substrate, and stepping up to more complex organisms, such as higher eukaryotes. Contributing to the building of such thorough descriptions of metal homeostasis is the challenge ahead in the field of Metals in Biology. However, for the time being, the general picture is that selectivity for the biological use of a given transition metal relies on its environmental availability, the cellular means to move it around as for muffling, the affinity of its cellular sensors and its protein hosts, and other cellular conditions triggering the need and use of metal ions. All of these aspects contribute together at the same time, without any clear dominance of one of them.

## 11. Conclusions and Perspectives

### 11.1. Metalloproteins: A Previous and Future Bonanza for Coordination Chemistry

**Figure 5:**
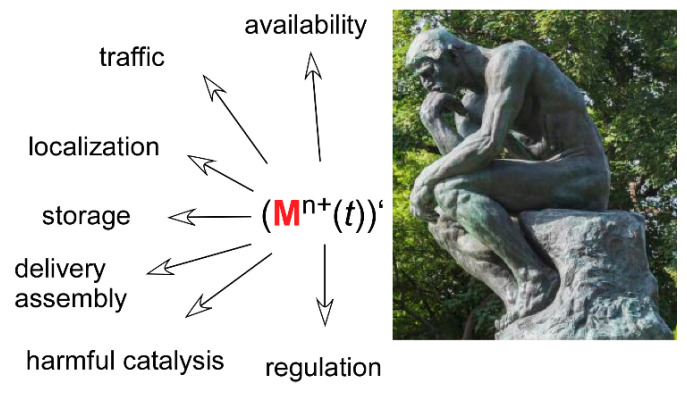
Transition metals in biology: a bonanza for coordination chemistry. ()’ stands for the derivative (Lagrange) notation with *t* being the time. The figure indicates some of the areas in which transition metal binding and exchange are, and will continue to be, studied. The picture is taken from http://www.musee-rodin.fr/fr/collections/sculptures/le-penseur.

The properties and reactivity of all ions of transition metals are relatively similar. Thus, cells deploy sophisticated means to select only one of them or for assembling relatively complex prosthetic groups when only one specific feature of a given metal is needed. This quest for the right metal in the right place is mandatory to avoid unwanted side reactions. Yet, the uniqueness of the catalyzed reaction/function–metal (group) relationship is not mandatory. In the microbial world, evolution managed to swap metals in some cases to adjust to their environmental availability. However, the recognition of a metal by the coordination sphere of a (would-be metallo) protein is not enough to guarantee selectivity in higher eukaryotes that do not have the genomic plasticity of microbes (and definitely less time and chances to develop it). Hence, metal exchange and channeling through particular pathways ensures metal homeostasis and, consequently, cell welfare. With only a few exceptions, these pathways have been detailed, or altogether discovered, over the last two decades only. This implies that a lot remains to be elucidated, including the interrelationship among the newly identified components. These biochemical reactions are inherently transient, which calls for methodological improvements tracking the metals inside cells, and hopefully later in organs and organisms, at the appropriate spatial and temporal resolutions. In this endeavor, Coordination Chemistry has a central place that is far more sophisticated than the mere definition of metal ligands and binding affinities. Indeed, the thermodynamic component of transition metal–protein interactions forms the basis upon which kinetic considerations will shape our understanding of metal homeostasis. With the legion of new methods, tools, concepts, and theoretical background that continuously develops, coordination chemistry will have to adjust to this moving landscape—a bonanza—and it will undoubtedly gain increased biological usefulness (Figure 5).

### 11.2. Metal Exchange: A Large Array of Applications in Biology

On the side of applications, the simplistic proposal that the toxicity of non-essential or of unduly abundant transition metals is by replacing essential ones at the active sites of enzymes and proteins will likely be superseded by subtler and probably more relevant descriptions. Indeed, metal replacement may make sense upon massive exposure (“shocks”) to one or several metals, but such acute conditions are relatively rare. Chronic low-level exposure instead instills the potential toxic metal with often resulting low intracellular concentrations. This process leverages a diversity of biochemical mechanisms that affect cellular functioning and lead to harm over time, as reviewed some time ago for one common metal toxicant [75,137]. Similarly, transition metal-related diseases are pathologies in which metal exchange on proteins is disturbed. Deeper analysis of the metal dynamics with the associated mechanistic insight will undoubtedly fuel innovative curative strategies and, possibly, will suggest efficient preventive measures.

### 11.3. Metals in Biology: A Plea for a Blended Flavor

**Figure 6:**
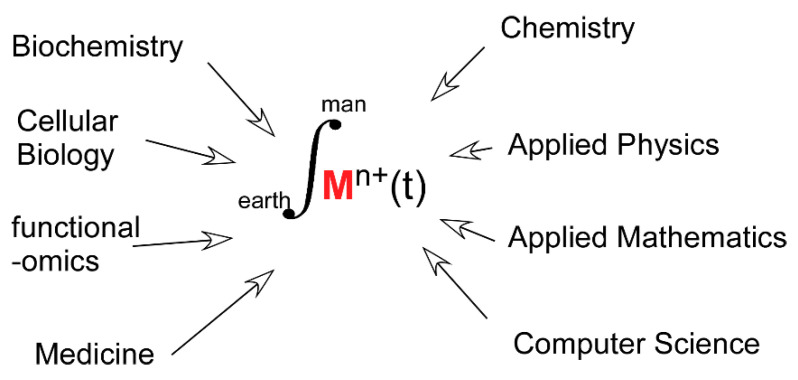
Transition Metals in Biology: a contemporary call for Integrative Science.

All the above conclusions have been obtained with a large diversity of experimental approaches and heterogeneous data sets. Forthcoming progress will rely on more quantitative measurements resulting from the use of more realistic biological models than the previously, and efficient, reductionist approaches that dominated the last four or five decades. This will lead to a shift in the boundaries of disciplines, such as Coordination Chemistry and “Inorganic” Biochemistry toward more biologically grounded subjects of studies. Yet, the benefit may be modest if the study of transition metals in biology remains the prerogative of specialists mastering a single method or a single experimental model. The time is now likely ripe for merging, rather than juxtaposing, disciplines contributing to the field. Investigations designed in this new way should lead to a higher level of understanding of the role played by the dynamics of transition metals exchange in proteins in a wealth of fundamental and applied biological questions (Figure 6).

## Figures and Tables

**Table 1:** Iron-sulfur stability in 2[4Fe-4S] ferredoxin from *Clostridium pasteurianum* ^1^.

Medium	% Replacement of [4Fe-4S] by [4Fe-4Se]	% Replacement of [4Fe-4Se] by [4Fe-4S]
No urea	0	22
8M urea	28	87

^1^ The fully loaded 2[4Fe-4S] (column 2) or 2[4Fe-4Se] (column 3) ferredoxin was anaerobically incubated 1 h at 25 °C with a molar excess of 80 moles/protein, i.e., 10x/cluster, of the other chalcogenide, ferrous iron, and dithiothreitol in Tris-Cl 0.1 M pH 8. The protein was separated from reactants on an anion-exchange column and the proportion of each chalcogenide-containing cluster was determined as previously described [101].
